# Enhanced Diagnosis of Pneumococcal Bacteremia Using Antigen- and Molecular-Based Tools on Blood Specimens in Mali and Thailand: A Prospective Surveillance Study

**DOI:** 10.4269/ajtmh.15-0431

**Published:** 2016-02-03

**Authors:** Jennifer C. Moïsi, Matthew Moore, Maria da Gloria Carvalho, Samba O. Sow, Duangkamon Siludjai, Maria Deloria Knoll, Milagritos Tapia, Henry C. Baggett, the Laboratory Evaluation of Assays for Pneumococcus (LEAP) Study Investigators

**Affiliations:** International Vaccine Access Center, Johns Hopkins Bloomberg School of Public Health, Baltimore, Maryland; Respiratory Diseases Branch, Centers for Disease Control and Prevention, Atlanta, Georgia; Centre pour les Vaccins en Développement, Bamako, Mali; Thailand Ministry of Public Health–U.S. Centers for Disease Control and Prevention Collaboration, Nonthaburi, Thailand; Division of Global Health Protection, Centers for Disease Control and Prevention, Atlanta, Georgia

## Abstract

Prior antibiotic use, contamination, limited blood volume, and processing delays reduce yield of blood cultures for detection of *Streptococcus pneumoniae*. We performed immunochromatographic testing (ICT) on broth from incubated blood culture bottles and real-time *lytA* polymerase chain reaction (PCR) on broth and whole blood and compared findings to blood culture in patients with suspected bacteremia. We selected 383 patients in Mali and 586 patients in Thailand based on their blood culture results: 75 and 31 were positive for pneumococcus, 100 and 162 were positive for other pathogens, and 208 and 403 were blood culture negative, respectively. ICT and PCR of blood culture broth were at least 87% sensitive and 97% specific compared with blood culture; whole blood PCR was 75–88% sensitive and 96–100% specific. Pneumococcal yields in children < 5 years of age increased from 2.9% to 10.7% in Mali with > 99% of additional cases detected by whole blood PCR, and from 0.07% to 5.1% in Thailand with two-thirds of additional cases identified by ICT. Compared with blood culture, ICT and *lytA* PCR on cultured broth were highly sensitive and specific but their ability to improve pneumococcal identification varied by site. Further studies of these tools are needed before widespread implementation.

## Introduction

Pneumonia is a leading cause of child morbidity and mortality worldwide.^1^,^2^ Clinical trials of pneumococcal conjugate vaccines (PCV) have shown that *Streptococcus pneumoniae* is responsible for 20–37% of chest X-ray positive pneumonia in children < 5 years of age.^3^–^5^ Etiological diagnosis of bacterial pneumonia relies primarily on blood culture that yields a positive result in 5–10% of hospitalized patients; a subset of these are identified as pneumococcus.^6^–^8^ Several factors reduce the sensitivity of blood culture, including small blood volumes, antibiotic pretreatment, specimen contamination by other organisms because of poor specimen collection or processing technique, prolonged transport time, and inconsistent availability of supplies. These factors are particularly common in resource-poor settings.^9^–^11^ However, even under optimal conditions, blood cultures identify only 30% of suspected cases of pneumococcal pneumonia,^5^,^12^ limiting the ability of surveillance to accurately estimate disease burden and evaluate the impact of vaccines.

The Binax NOW^®^ antigen assay (Alere, Waltham, MA) for *S. pneumoniae* is a rapid immunochromatographic test (ICT) licensed for testing of urine from adult pneumonia patients and of cerebrospinal fluid (CSF) from meningitis patients of all ages.^13^ In children, the Binax ICT has limited value for testing urine, as it cannot distinguish pneumococcal pneumonia from nasopharyngeal (NP) colonization.^14^–^19^ In contrast, the test is 90–100% sensitive and specific when testing CSF from meningitis patients of all ages compared with culture, latex agglutination, and polymerase chain reaction (PCR). ICT also enables detection of a large number of pneumococcal meningitis cases among patients with negative CSF results.^20^–^23^ Recent data suggest that ICT may also be used to identify pneumococcus from blood specimens that indicate bacterial growth after incubation in automated culture systems but fail to yield an organism on subculture.^24^–^26^

Molecular methods such as PCR may further enhance pneumococcal detection in patients with suspected invasive pneumococcal disease (IPD).^27^–^31^ Moreover, multiplex conventional and real-time PCR can be used to identify serotype-specific capsular genes in clinical specimens,^32^,^33^ providing valuable data on the serotype distribution of culture-negative specimens.^34^,^35^ However, it is uncertain whether a positive real-time *lytA* PCR result from blood can be triggered by colonization alone^36^,^37^ (PERCH presentation ISPPD 2014).

National policies regarding the use of PCVs are based, in part, on disease burden estimates. The challenge of obtaining these data from low- and middle-income countries where the burden of pneumococcal disease is thought to be very high further emphasizes the urgency of better diagnostics. The objective of the Laboratory Evaluation of Assays for Pneumococcus (LEAP) study was to assess the performance of the Binax NOW ICT on blood culture broth and of real-time PCR for *lytA* on whole blood and blood culture broth for the diagnosis of pneumococcal bacteremia in Thailand and Mali.

## Methods

### Patient enrollment and specimen collection.

This study was nested in existing IPD surveillance projects in Mali and Thailand (Table 1). In Mali, all inpatients 0–15 years and outpatients 0–35 months of age presenting to Hôpital Gabriel Touré, the main pediatric referral hospital in Bamako, with fever ≥ 39°C or a suspicion of meningitis, pneumonia, or sepsis and providing informed consent were eligible for inclusion in this study. Study participants had blood collected and inoculated into a blood culture bottle for immediate processing at the hospital microbiology laboratory. Blood collected concurrently with blood cultures was also processed, and serum and whole blood were stored frozen at −80°C until further testing. In Thailand, blood cultures were collected as clinically indicated from hospitalized patients of all ages identified at the 18 district hospitals and two provincial hospitals in Sa Kaeo and Nakhon Phanom provinces and processed at the provincial hospital laboratory as part of routine surveillance. Cultures collected at district hospitals were maintained at 15–30°C and transported within 24 hours to the provincial hospitals for processing. Patients who consented to participate in a related study on respiratory pathogens also provided whole blood and NP swabs, but these were usually obtained at a different time than blood culture specimens, often after initiation of antimicrobial treatment. Whole blood and NP specimens collected at district hospitals were transported to the provincial hospitals where they were frozen at −70°C before shipping on dry ice to Bangkok for testing. Whole blood specimens were stored on ice packs for a median of 26 hours (interquartile range [IQR]: 19–36 hours) before freezing. At both sites, a filter paper disc was saturated with 20 μL of patient serum and frozen before testing for the presence of antimicrobial activity.

### Laboratory methods.

All blood cultures were processed using automated culture systems: the BACTEC 9060 system (Beckton-Dickinson, Sparks, MD) was used in Mali and the BacT/ALERT 3D system (bioMerieux, Hazelwood, MI) was used in Thailand. Cultures with a positive signal in the automated system (i.e., alarm-positive cultures) were subcultured by standard methods.

Aliquots of blood culture broth from included patients were frozen between −80°C and −70°C for batch testing by ICT and PCR. Serum was tested for the presence of antimicrobial agents by assessing its ability to inhibit growth of a pan-sensitive *Staphylococcus aureus* ATCC 25923 strain, as described previously.^11^

ICT was performed on blood culture specimens after thawing to room temperature according to the manufacturer's instructions for testing urine and CSF. The presence of a control line was required to confirm test validity. In Mali, specimens were categorized as positive or negative based on the presence or absence of a test line. In Thailand, previous studies had shown that ICT results can sometimes be equivocal,^25^ so we developed a scoring system a priori according to the intensity of the color of the test line. Specimens with a “very strong” or “strong” test line were considered positive while those with a “weak” or absent test line were considered negative. A single technician was responsible for conducting the tests at each site, averting any inter-rater variability in interpretation.

Technicians from both sites were trained to conduct real-time *lytA* PCR and sequential multiplex serotyping PCR on blood culture broth and whole blood specimens at the Centers for Disease Control and Prevention *Streptococcus* Laboratory in Atlanta, GA, using the first 25% of study specimens and then completed all remaining testing in-country. Real-time PCR for pneumococcus (*lytA* gene) detection was performed as previously described for all specimens included in this study.^28^ In brief, 200 μL of whole blood ethylenediaminetetraacetic acid (EDTA) or 50 μL of blood cultured broth was added to 100 μL of Tris-EDTA buffer containing 0.04 g/mL lysozyme and 75 U/mL mutanolysin (Sigma Chemical Co., St. Louis, MO), and the mixture was incubated for 1 hour at 37°C. DNA extraction was performed by following Qiagen DNA Mini Kit instructions (Qiagen, Venlo, The Netherlands). DNA extracted from blood cultured broth was diluted to 1:100 to1:1,000 to avoid PCR inhibition often observed from specimens with extremely high pneumococcal DNA concentrations. A whole blood EDTA sample was considered positive if its cycle threshold (Ct) value was ≤ 35 and negative if its Ct value was > 40. If a Ct value was > 35 and ≤ 40, the specimen was diluted 10-fold and retested to determine whether PCR inhibitors were present. The specimen was considered positive if the Ct value of the diluted specimen was ≤ 35, equivocal if the Ct value was 36–40, and negative if > 40. Multiplex real-time PCR for pneumococcal serotyping was performed for *lytA*-positive whole blood and NP specimens,^38^,^39^ whereas conventional multiplex PCR was used to serotype *lytA*-positive incubated blood cultured broth.^33^ A subset of specimens from both sites was sent to Atlanta for quality control testing.

### Selection of specimens for testing and analysis.

Patients were selected for inclusion into the LEAP study based on their automated blood culture and subculture results, according to four groups:

• Group 1: Blood culture positive for *S. pneumoniae* (positive controls).
• Group 2: Blood culture positive for another pathogen (negative controls); this included patients culture positive for any *Streptococcus* species other than *S. pneumoniae* and for non-*Streptococcus* pathogens.
• Group 3: Alarm positive after incubation in an automated blood culture system, subculture negative.
• Group 4: Alarm negative.

### Statistical methods.

Before enrollment began, sample sizes sufficient to achieve the study objectives were estimated using simulation. On the basis of the assumed conditional probabilities relating the tests to each other, latent class analysis (LCA) was used to determine adequate sample sizes. The simulation and LCA were conducted in SAS Version 9.3 (SAS Institute Inc., Cary, NC). The resultant target sample sizes for Mali were 75 for group 1, 100 for group 2, 20 for group 3, and 200 for group 4. The corresponding targets for Thailand were 35, 100, 100, and 200, respectively. In Mali, if the total number of specimens available for any given group exceeded the target, we used random sampling to achieve the target number. In Thailand, sampling was only done for group 4.

### Data analysis.

We estimated the sensitivity and specificity of the ICT and *lytA* real-time PCR based on results from culture-positive patients (groups 1 and 2) and the potential additional pneumococcal yield achieved by testing culture-negative cases (groups 3 and 4), stratified by study site, age, clinical syndrome, antibiotic pretreatment, and NP pneumococcal carriage status when available. Finally, for children < 5 years of age, we calculated the increased pneumococcal yield that could be achieved by adding each of the new diagnostics to blood culture as follows:

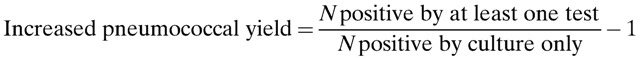


To estimate the overall potential added yield of ICT and PCR in these surveillance settings, we assumed that the proportion positive among all blood culture–negative patients was equal to the proportion observed in the sample of blood culture–negative patients who were tested in this study.

Analyses were conducted in Stata 12 (Stata Corp., College Station, TX) and Microsoft Excel (Microsoft Corp., Redmond, WA).

### Ethical considerations.

This study was conducted in accordance with the ethical principles for human subjects research of the Declaration of Helsinki and received approval from the Institutional Review Boards of the Johns Hopkins Bloomberg School of Public Health, University of Maryland School of Medicine, Centers for Disease Control and Prevention, and University of Bamako Faculty of Medicine, Pharmacy and Dentistry and from the Ethical Committee for Human Subjects Research of the Thailand Ministry of Public Health.

## Results

From a total of 5,086 patient blood cultures obtained in Mali between January 25, 2010 and January 24, 2011, we selected 383 (7.5% of total, 90.9% < 5 years of age) for inclusion based on their blood culture findings: 75 positive controls (group 1), 100 negative controls (group 2), 10 alarm-positive, subculture-negative cases (group 3), and 198 alarm-negative cases (group 4). In Thailand, 596 (4.1%) of 14,429 blood cultures collected during May 2010 to April 2011 were included, of which 39.4% were from patients < 5 years of age: 31 positive controls (group 1), 162 negative controls (group 2), 183 alarm-positive, subculture-negative cases (group 3), and 220 alarm-negative cases (group 4). We were unable to achieve our target numbers of alarm-positive, subculture-negative patients in Mali and culture-confirmed pneumococcal cases in Thailand because of limited numbers of patients in each of these groups. Patient characteristics are detailed in Table 2. In Mali, most participants were young children and nearly half had meningitis, in contrast with Thailand where a majority were adults with pneumonia. At both sites, patients with positive cultures were less likely to have had antibiotics before culture collection (*P* = 0.03). In Mali, patients with pneumococcal bacteremia were younger than the other three inclusion groups (*P* < 0.01). In Thailand, pneumococcal carriage among those tested was found in 88% of eight positive controls (all ≥ 5 years of age), 42% of 213 alarm-negative patients (all < 5 years of age), and approximately 12% of the other two groups (44 and 16 tested for groups 2 and 3, of which 3 and 2 were < 5 years of age) (*P* < 0.01).

Table 3 shows ICT findings by site and study group. In Mali, ICT sensitivity and specificity compared with blood culture were both 100% (one-sided 95% confidence interval [CI]: 95.2–100% and 96.4–100%, respectively). In Thailand, sensitivity and specificity among all ages were 87.1% (95% CI: 70.1–96.3%) and 96.9% (95% CI: 92.9–99.0%), respectively (Table 3); among children < 5 years of age, sensitivity (based on only two cases of pneumococcal bacteremia) and specificity (based on nine cases of non-pneumococcal bacteremia) were 50% (95% CI: 1.3–98.7%) and 89% (95% CI: 51.7–99.7%), respectively. All five negative controls in Thailand with a positive ICT result had a *Streptococcus* species other than *S. pneumoniae* identified by culture (three alpha-hemolytic viridans-like streptococci and two Group A streptococci, of which one also had *Shigella flexneri* detected). Of note, an additional 115 non-pneumococcal streptococci had a negative ICT result in Thailand; the negative control group in Mali did not include any patients that were culture positive for non-pneumococcal streptococci. In Mali, ICT was negative for all 208 patients that were blood culture negative (groups 3 and 4). In Thailand, 10 of 403 (2.5%) blood culture–negative cases were ICT positive, mostly among the alarm-negative subgroup (group 4): nine of 220 (4.1%) compared with one of 183 (0.55%) alarm-positive, subculture-negative (group 3) cases. Of the 10, eight were from children < 5 years of age, four had an admission diagnosis of pneumonia and six of an other syndrome, and two had documented pneumococcal NP colonization (among eight tested); none had serum antibiotic results available.

Real-time PCR on blood culture broth compared with culture was 100% (one-sided 95% CI: 96.4–100%) sensitive and 98.9% (95% CI: 93.6–100%) specific in Mali and 90.3% (95% CI: 74.2–98.0%) sensitive and 100% (one-sided 95% CI: 97.7–100%) specific in Thailand (Table 4). PCR on cultured broth did not yield any additional positives among blood culture–negative patients at either site. Quality control of PCR on blood culture broth produced identical results to initial testing for both sites (29 positive and four negative specimens tested in Mali, 10 positive and 33 negative specimens tested in Thailand).

Whole blood was available for PCR testing from 381 (99%) patients in Mali and 228 (38%) in Thailand (Table 5). In Mali, real-time PCR on whole blood was 87.8% (95% CI: 78.2–94.3%) sensitive and 96.0% (95% CI: 90.0–98.9%) specific (positive in one each of *Haemophilus influenzae* type b, *Klebsiella pneumoniae*, *S. aureus*, and group D *Salmonella*). On quality control testing, eight of 13 positive controls and one of two negative controls that had originally tested positive were found negative by real-time PCR. Because thawing of specimens during transport may have degraded DNA, we used only the original testing results in the final analysis. In Thailand, real-time PCR on whole blood was 37.5% (95% CI: 8.5–75.5%) sensitive (based on the eight culture-confirmed pneumococcal cases with blood specimens available) and 100% (one-sided 95% CI: 91.4–100%) specific from initial testing, with sensitivity increasing to 75.0% (95% CI: 34.9–96.8%) after quality control testing (three new positives identified) and specificity reduced (one positive of two negative controls tested). Fifteen patients were positive and three equivocal by whole blood PCR among 207 culture-negative patients in Mali (8.7% yield) compared with only three positives among 179 tested in Thailand (1.7% yield); a fourth positive was detected during quality control testing of 42 culture-negative, whole blood PCR–negative specimens in Thailand, increasing the yield to 2.2%. In the culture-negative group, quality control testing found nine negatives among 12 PCR-positive specimens in Mali and one negative of two PCR positives in Thailand. Again, because of the possibility of DNA degradation during transport, these quality control results were not considered in the final analysis.

**Figure 1.**
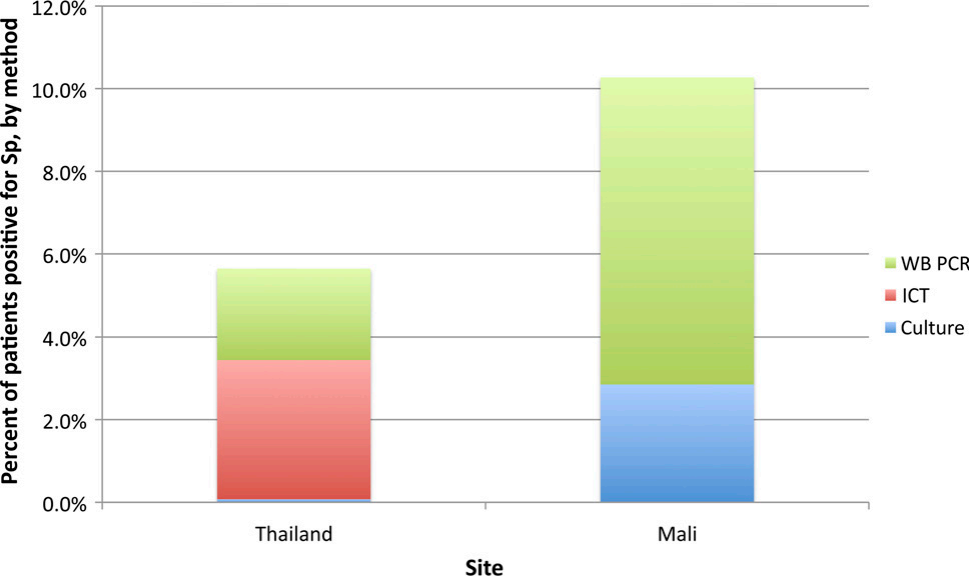
Additional pneumococcal yield with immunochromatographic testing of blood culture broth and *lytA* polymerase chain reaction (PCR) of whole blood.

We estimated the potential increase in pneumococcal yield that could be achieved in children < 5 years of age if all patients who had a blood culture were tested by ICT and real-time PCR according to our protocol, based on the sampling fraction that was applied when selecting specimens for each group (Figure 1

). In Mali, pneumococcal yield increased from 2.9% by culture alone to 10.3% with the addition of *lytA* real-time PCR on whole blood; the other two tests did not improve yield further. In Thailand, yield by culture alone was 0.1%, increasing to 3.4% with the addition of the ICT and 5.6% with whole blood PCR. Because ICT and whole blood PCR contributed differently to yield in the two sites, there was insufficient crossover to successfully estimate sensitivity and specificity using latent class analysis.

Serotyping by multiplex conventional or real-time PCR yielded a serotype in 73 of 76 blood culture broth and 50 of 86 whole blood specimens that were positive or equivocal for *lytA* in Mali, and in 28 of 28 broth and eight of 10 whole blood specimens in Thailand. In Mali, results were concordant for 46 of 65 patients with serotyping attempted on both specimens (the remainder had one typable and one non-typable specimen; none yielded two different known serotypes) compared with four of six patients in Thailand (the other two were 19A from broth/non-typable from whole blood and 19A from broth/6 from whole blood). None of the four negative controls from Mali with a positive whole blood PCR result had a serotype identified from whole blood (one not tested, three non-typable).

## Discussion

In this study, we aimed to assess the performance and potential contribution of the *S. pneumoniae* ICT and real-time PCR for identification of cases of pneumococcal bacteremia. We implemented the project in sites in Mali and Thailand alongside ongoing surveillance projects that obtained blood for culture from well-characterized populations, and selected patients based on their blood culture results to constitute a positive control group, a negative control group, and a target group for assessing increased detection of pneumococcal bacteremia. In Mali, the ICT and real-time PCR were highly sensitive and specific compared with blood culture, and real-time PCR on whole blood identified possible pneumococcal cases among culture-negative patients, producing a > 3-fold increase in yield compared with culture alone in children < 5 years of age, from 2.9% to 10.3%. In Thailand, all three tests showed good sensitivity and specificity, and both the ICT and the real-time PCR on whole blood detected possible pneumococcal cases in the culture-negative group, increasing the overall yield in children < 5 years from < 0.1% to 5.6%.

The differences in findings between Mali and Thailand are challenging to interpret. Patient populations differed greatly between sites in terms of age, clinical syndrome, and hospitalization status. Prior antibiotic use was similar based on serum disc test results, but half of all patients in Thailand had no specimen available for testing, leading to potential selection bias. Blood cultures were collected systematically for all patients meeting specific inclusion criteria in Mali, but only at clinicians' discretion in Thailand. Whole blood was collected concurrently with blood cultures in Mali. Conversely, in Thailand whole blood was available only for patients enrolled in a separate study and was often collected up to 24 hours after blood culture; antibiotic exposure before whole blood collection was therefore very likely even if it had not occurred before culture, potentially affecting sensitivity of PCR on whole blood and limiting additional case detection. Blood was immediately transported to the on-site laboratory and processed or frozen for later testing in Mali, whereas several hours of transport time were required from the sites of specimen collection to the laboratory in Thailand (for whole blood, median time from collection to freezing: 26 hours, IQR: 19–36 hours). Different automated blood culture machines and broth bottles were used, possibly affecting ICT results: in another study in Thailand, weakly positive ICT results were seen among uninoculated BacTAlert bottles but not on BACTEC aerobic bottles, pointing to potential cross-reactivity with the BacTAlert system.^25^ Longer specimen transport times in Thailand and different blood culture systems may have contributed to the higher numbers of alarm-positive cultures that were subculture negative compared with Mali. Different age distributions may also have been a factor, as a higher percentage of specimens from patients aged 5–14 years in both sites were alarm positive, subculture negative. Finally, in Thailand, many patients who should have had a whole blood specimen available for testing did not: this limited the sample size available for the whole blood PCR analysis and potentially caused selection bias. It is therefore likely that the tests improved ascertainment of pneumococcal disease differently across sites because of differences in patient populations and methodology. It is possible that if we had sufficient sample size to compare results across similar strata (in particular age, clinical syndrome, and prior antibiotic use), we would obtain similar results in terms of sensitivity, specificity and additional pneumococcal yield.

The ICT on blood culture broth performed well in both sites in terms of sensitivity and specificity compared with culture, with false positives seen in Thailand in three patients with blood cultures positive for a *Streptococcus* species known to cross-react with the ICT and in two patients with group A Streptococcal bacteremia.^13^ Additional possible pneumococcal cases identified by ICT were substantial in Thailand and concentrated in children < 5 years. Only one of these additional cases was detected among the alarm-positive, subculture-negative group, which was unexpected and does not support the hypothesis that alarm-positive subculture-negative cultures result from pneumococcal infections that fail to grow on subculture, as suggested in previous work from Thailand.^25^ ICT detected no additional cases in Mali. Our findings are similar to those from a study evaluating the ICT in CSF, in which the ICT performed significantly better than culture and latex agglutination in two Asian sites but similar to these assays in three African sites.^23^ Too few patients in Thailand had serum antibiotic data available to determine whether pretreatment affected the relative yields of ICT and culture. There was no difference in ICT yields among culture-negative patients by NP carriage status, suggesting that cases identified only by ICT likely represent true cases rather than false positives caused by colonizing isolates or antigens entering the bloodstream.

Real-time *lytA* PCR on blood cultured broth was highly sensitive and specific relative to blood culture, did not detect any pneumococci among culture-negative patients, and was consistent in field and quality control testing. In particular, none of the culture-negative, ICT-positive cases were positive by PCR on blood cultured broth; if these cases are truly pneumococcal, this may point to a technical issue with broth PCR.

On whole blood, real-time PCR specificity was high in both sites and sensitivity ranged from 75% in Thailand to nearly 90% in Mali. The number of specimens tested in Thailand was low, and maximum sensitivity was only achieved by considering positive results from the local laboratory and those from subsequent quality control testing overseen by a senior laboratory scientist. It appears that many factors can affect whole blood PCR results, including antibiotic pretreatment, processing delays, testing delays, extraction methods, repeated freeze-thaw cycles, and technician skill, with regular training and quality control in an expert laboratory necessary to achieve optimal results. Despite these limitations, whole blood PCR produced a substantial increase in pneumococcal yield over culture alone in children < 5 years of age in both sites (but a smaller increase over culture and ICT combined in Thailand). The absolute increase in yield from whole blood PCR was of the same order of magnitude for the two sites (+2.2% to +7.4%), and the final pneumococcal prevalence accounting for ICT and PCR positives was 5.6% in Thailand and 10.2% in Mali. Several observations suggest that many culture-negative, whole blood PCR–positive cases may be true pneumococcal cases. The great majority of them were children < 5 years of age (16 of 18 in Mali and three of three in Thailand) who were more likely to have false-negative blood cultures because of low blood volumes. They appeared more likely to have pre-culture antibiotic treatment than the general surveillance population (five of 18 or 28% in Mali versus 17% among all subjects, one of one tested in Thailand), which may have inhibited pneumococcal growth on culture. On the other hand, all had negative ICT results on culture broth and few were alarm positive (one in Mali, none in Thailand), which runs counter to the hypothesis that cultured pneumococci underwent autolysis prior to subculture. Given their age, they were also more likely to be pneumococcal carriers, and therefore more likely to have a false-positive *lytA* PCR result. Finally, our lack of a healthy control group limits our ability to attribute disease in these patients to pneumococcus. The detection of *lytA* in patients with blood cultures positive for other pathogens supports that false-positive PCR results can occur, suggesting that PCR-positive whole blood may be seen in the absence of pneumococcal disease.

## Conclusion

Clinical trials of pneumococcal conjugate vaccines have demonstrated the poor sensitivity of blood culture for diagnosing pneumococcal pneumonia,^5^,^12^ and numerous studies suggest that its sensitivity may be lower in Asia than in Africa because of widespread pre-culture antibiotic use. The use of novel tools such as the ICT or real-time PCR on blood specimens may increase case detection, help improve disease burden estimates, and facilitate evaluations of PCV impact. Our study has shown that the amount of benefit from these additional assays may vary across settings; the decision to use either of these tools should be made with careful consideration of specific objectives and of population characteristics.

## Figures and Tables

**Table 1:** LEAP study methods

	Thailand	Mali
Hospitals	Two provincial hospitals and 18 district hospitals in Sa Kaeo and Nakhon Phanom provinces	Hôpital Gabriel Touré, the largest pediatric hospital in Bamako, the capital city
Inclusion criteria note: specimens from only a subset of these patients were selected, as determined by the blood culture results	Persons of all ages hospitalized with suspicion of pneumonia and with a blood culture obtained	Children < 16 years of age hospitalized with fever (≥ 39°C) and/or suspicion of invasive bacterial infection and with a blood culture obtained
	Children < 5 years of age hospitalized with suspicion of sepsis and with a blood culture obtained	Children < 36 months of age treated as outpatient with fever (≥ 39°C) and/or suspicion of invasive bacterial infection and with a blood culture obtained
	Note: blood cultures obtained at clinician discretion	
Exclusion criteria	None	Newborns who have not yet been discharged from the hospital
Informed consent	Informed consent waiver granted	Required
	Subset of patients enrolled in a separate pneumonia etiology study (RPS) consented to sharing information with this study	
Blood volume (target)	Culture: 4 mL for < 5-year-olds, 10 mL for all other ages	Culture: 1 mL for < 1 month, 2 mL for 1 month to 4 years, 3 mL for > 4-year-olds
	Whole blood: 2 mL for PCR (0.2 mL per single PCR run; RPS subjects only)	Whole blood: 2 mL for PCR (0.2 mL per single PCR run)
	Serum: 0.5 mL for antimicrobial activity testing	Serum: 0.5 mL for antimicrobial activity testing
NP swabs	Yes for RPS patients	No
Specimen transport procedures	Blood culture bottle placed in automated blood culture machine and blood transported to laboratory within 4 hours for serum separation	Blood culture bottle placed in automated blood culture machine and serum transported to laboratory within 1 hour
	Blood for PCR and NP specimens collected after blood for cultures, stored on ice until frozen at −70°C at provincial hospital and then shipped on dry ice to Bangkok for testing	Blood for PCR and serum collected simultaneously with blood for culture, frozen at −80°C within 24 hours of collection

LEAP = laboratory evaluation of assays for *Pneumococcus*; NP = nasopharyngeal; PCR = polymerase chain reaction; RPS = respiratory pathogen study.

**Table 2:** Patient characteristics by study site

Characteristic	Thailand (*N* = 596)								Mali (*N* = 383)							
	Group 1 (*N* = 31)		Group 2 (*N* = 162)		Group 3 (*N* = 183)		Group 4 (*N* = 220)		Group 1 (*N* = 75)		Group 2 (*N* = 100)		Group 3 (*N* = 10)		Group 4 (*N* = 198)	
	*n*	%	*n*	%	*n*	%	*n*	%	*n*	%	*n*	%	*n*	%	*n*	%
Age (years)	*P* = 0.39*								*P* < 0.01							
< 1	1	3.2	5	3.1	7	3.8	69	31.4	42	56.0	41	41.0	5	50.0	104	52.5
1–4	1	3.2	4	2.5	0	0.0	144	65.5	26	34.7	44	44.0	0	0.0	86	43.4
5–14	2	6.5	3	1.9	8	4.4	0	0.0	7	9.3	15	15.0	5	50.0	8	4.0
15–64	16	51.6	89	54.9	108	59.0	5	2.3	–	–	–	–	–	–	–	–
65 +	11	35.5	61	37.7	60	32.8	2	0.9	–	–	–	–	–	–	–	–
Sex	*P* = 0.48								*P* = 0.67							
Male	21	67.7	98	60.5	100	54.7	129	58.6	42	56.0	55	55.0	5	50.0	121	61.1
Diagnosis†	*P* < 0.01								*P* = 0.09							
Meningitis	1	3.2	9	5.6	1	0.6	18	8.2	38	50.7	37	37.0	5	50.0	89	45.0
Pneumonia w/ pleural effusion	0	0.0	2	1.2	2	1.1	1	0.5	2	2.7	3	3.0	1	10.0	0	0.0
Pneumonia w/o pleural effusion	15	48.4	23	14.2	36	19.7	153	69.6	20	26.7	23	23.0	2	20.0	59	29.8
Sepsis	6	19.4	69	42.6	47	25.7	20	9.1	0	0.0	2	2.0	0	0.0	2	1.0
Other	9	29.0	59	36.4	97	53.0	28	12.7	15	20.0	35	35.0	2	20.0	48	24.2
Prior antibiotics‡	*P* = 0.03								*P* = 0.03							
Yes	0	0.0	8	8.8	24	20.0	9	13.0	6	8.0	15	15.0	2	20.0	45	22.7
No	19	100.0	83	91.2	96	80.0	60	87.0	69	92.0	85	85.0	8	80.0	153	77.3
NP carriage	*P* < 0.01															
Yes	7	87.5	5	11.4	2	12.5	90	42.3	–	–	–	–	–	–	–	–
No	1	12.5	39	88.6	14	87.5	123	57.8	–	–	–	–	–	–	–	–

Group 1: culture positive for *Streptococcus pneumoniae*; Group 2: culture positive for other pathogen; Group 3: alarm positive, subculture negative; Group 4: alarm negative. *P* values calculated for comparison among groups within each site.

* *P* value comparing age distribution in groups 1–3, excluding group 4 cases that were selected among children < 5 years of age (except for seven patient enrollment errors).

† Clinician's diagnosis at time of enrollment; “other” patients in Thailand had a range of syndromes including febrile illness without a focus, urinary tract infections, upper respiratory infections, acute exacerbation of chronic obstructive pulmonary disease, heart disease, injury, and so on; “other” patients in Mali had febrile illness without a focus (72 outpatients) or other suspected invasive disease (osteomyelitis, septic arthritis, endocarditis, etc.).

‡ Prior antibiotic use based on serum disc antimicrobial activity test result.

**Table 3:** Binax ICT results on blood culture broth specimens according to blood culture result

Binax ICT result	Thailand (*N* = 596)								Mali (*N* = 383)							
	Group 1		Group 2		Group 3		Group 4		Group 1		Group 2		Group 3		Group 4	
	All	< 5 years	All	< 5 years	All	< 5 years	All	< 5 years	All	< 5 years	All	< 5 years	All	< 5 years	All	< 5 years
Positive	27 (87)	1 (50)	5 (3)*	1 (11)	1 (0.5)	0 (0)	9 (4)	8 (4)	75 (100)	68 (100)	0 (0)	0 (0)	0 (0)	0 (0)	0 (0)	0 (0)
Negative	4 (13)	1 (50)	157 (97)	8 (89)	182 (99.5)	7 (100)	211 (96)	205 (96)	0 (0)	0 (0)	100 (100)	85 (100)	10 (100)	5 (100)	198 (100)	190 (100)
Total	31	2	162	9	183	7	220	213	75	68	100	85	10	5	198	190

ICT = immunochromatographic test.

Number (%) with positive or negative ICT result is shown by age and blood culture result.

* All five negative controls with a positive ICT result had a *Streptococcus* species other than *Streptococcus pneumoniae* identified by culture (in Mali, none of the negative controls used to estimate specificity had blood cultures positive for non-pneumococcal streptococci).

**Table 4:** *lytA* real-time PCR testing on blood culture broth specimens according to blood culture result

Blood cultured broth PCR	Thailand (*N* = 596)								Mali (*N* = 383)							
	Group 1		Group 2		Group 3		Group 4*		Group 1		Group 2		Group 3		Group 4	
	All	< 5 years	All	< 5 years	All	< 5 years	All	< 5 years	All	< 5 years	All	< 5 years	All	< 5 years	All	< 5 years
Positive	28 (90)	1 (50)	0 (0)	0 (0)	0 (0)	0 (0)	0 (0)	0 (0)	75 (100)	68 (100)	1 (1)	1 (1)	0 (0)	0 (0)	ND	ND
Negative	3 (10)	1 (50)	162 (100)	9 (100)	183 (100)	7 (100)	220 (100)	213 (100)	0 (0)	0 (0)	99 (99)	84 (99)	10 (100)	5 (100)	ND	ND
Total	31	2	162	9	183	7	220	213	75	68	100	85	10	5	ND	ND

PCR = polymerase chain reaction; ND = not done.

Number (%) with positive or negative real-time PCR result on blood culture broth is shown by age and blood culture result.

* Protocol did not require PCR testing on these specimens.

**Table 5:** *lytA* real-time PCR testing on whole blood specimens according to blood culture results

Whole blood PCR	Thailand (*N* = 596)								Mali (*N* = 383)							
	Group 1		Group 2		Group 3		Group 4		Group 1		Group 2		Group 3		Group 4	
	All	< 5 years	All	< 5 years	All	< 5 years	All	< 5 years	All	< 5 years	All	< 5 years	All	< 5 years	All	< 5 years
Positive	6 (75)	0	0 (0)	0 (0)	0 (0)	0 (0)	4 (2)	4 (2)	65 (88)	58 (87)	4 (4)	4 (5)	1 (10)	0 (0)	17 (9)	16 (8)
Negative	2 (25)	0	41 (100)	2 (100)	16 (100)	2 (100)	159 (98)	159 (98)	9 (12)	9 (13)	96 (96)	81 (95)	9 (90)	5 (100)	180 (91)	173 (92)
Total	8	0	41	2	16	2	163	163	74	67	100	85	10	5	197	189

PCR = polymerase chain reaction.

Number (%) with positive or negative real-time PCR result on whole blood is shown by age and blood culture result.
